# Constrictive Pericarditis as an Initial Manifestation of Systemic Lupus Erythematosus

**DOI:** 10.7759/cureus.11256

**Published:** 2020-10-30

**Authors:** Samiksha Gupta, Gautam Jesrani, Saurabh Gaba, Monica Gupta, Suraj Kumar

**Affiliations:** 1 General Medicine, Government Medical College and Hospital, Chandigarh, IND; 2 Cardiology, Government Medical College and Hospital, Chandigarh, IND

**Keywords:** sle, lupus, cardiac, constrictictive pericarditis, systemic lupus erythematosus

## Abstract

A young female presented with new-onset rash, oral ulcers and dyspnea without overt features of heart failure. She was diagnosed with systemic lupus erythematosus with early constrictive pericarditis, cutaneous lupus and serositis in the form of pericardial and pleural effusion. There was no renal, neurological and joint involvement. She was treated with steroid pulse and other ancillary drugs that led to remission with improvement in the symptoms and reversal of echocardiographic changes of constrictive pericarditis. Oral steroids were successfully tapered off after four months, and only hydroxychloroquine was continued. Constrictive pericarditis is an uncommon feature of lupus and its occurrence as an initial manifestation, without a history of repeated episodes of acute pericarditis, is rarely reported.

## Introduction

Constrictive pericarditis (CP) refers to fibrosis of the pericardium secondary to an inflammatory disease process. The scarred and stiff pericardium loses its elasticity, and it fails to expand in conjunction with the right ventricular distension during diastole. This leads to impaired right ventricular filling with bulging of the interventricular septum into the left ventricle (ventricular interdependence) [1]. The diastolic dysfunction is eventually accompanied by decreased left ventricular volume and reduced stroke volume. The clinical features include dyspnea, fatigue, peripheral edema, ascites and pulmonary edema with accompanying orthopnea and paroxysmal nocturnal dyspnea. The vital clinical signs are elevated jugular venous pressure (JVP) that may fail to decline during inspiration (Kussmaul’s sign) and an exaggerated fall of more than 10 mm of Hg in systolic blood pressure during inspiration (pulsus paradoxus) [2]. A pericardial knock may be audible on auscultation of the heart. Advanced disease is characterized by marked weight loss with muscle wasting (cachexia). While tuberculosis is the chief cause in developing countries, idiopathic or viral etiology is the most common cause in developed countries [1]. The other causes include cardiac surgery, radiotherapy, malignancies, chronic kidney disease, drugs, pyogenic pericarditis, trauma and connective tissue disorders. CP often presents with an obscure etiology. A thorough history and physical examination with an extensive panel of investigations, including pericardial biopsy, may be needed to ascertain the cause, which may never be established in some cases. In this report, a case of systemic lupus erythematosus (SLE) having early CP at the index presentation is described.

## Case presentation

**Figure 1:**
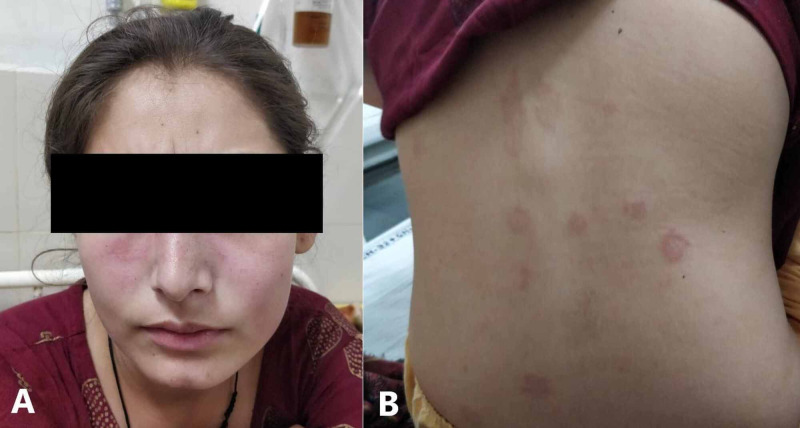
A) Erythematous and non-blanching malar rash on the face with nasolabial sparing. B) Rash with similar characteristics on the back.

A 20-year-old female presented with fatigue, low-grade fever, painless oral ulcers, a rash on face and back (Figure 1), and gradually progressive shortness of breath with dry cough for one month. There was no history of orthopnea, paroxysmal nocturnal dyspnea, palpitations and swelling of lower limbs. There was no significant history, and she did not report having alopecia, photosensitivity, joint pains or stiffness, blue discolouration of fingers, dry eyes and mouth, tightening of skin or difficulty in swallowing. She had a blood pressure of 110/70 mm of Hg with a regular and good volume pulse, and JVP was elevated. Investigations revealed mild thrombocytopenia and lymphopenia with normal haemoglobin. Renal and liver function tests were normal, and urinary sediment was bland without significant proteinuria. An electrocardiogram was suggestive of sinus tachycardia, and there was no abnormality on chest radiograph and ultrasound of the abdomen. Specialized tests (Table 1) confirmed the diagnosis of a lupus flare with absent antiphospholipid antibodies and cryoglobulins. The inflammatory markers and autoimmune antibodies were elevated, and serum complement level was reduced. The blood and urine cultures were sterile, and procalcitonin level was normal.

**Table 1:** Special investigations panel that confirmed diagnosis of a lupus flare. ESR, erythrocyte sedimentation rate; CRP, C-reactive protein; RF, rheumatoid factor; ANA, antinuclear antibody; Anti-dsDNA, anti-double stranded deoxyribonucleic acid antibody; Anti-Sm, anti-smith antibody

Investigation	Value	Normal range
ESR (mm/hr)	46	0-20
CRP (mg/L)	39	<5
RF (U/mL)	14	<10
ANA (titer)	1:1280	<1:40
Anti-dsDNA (U/mL)	178	<30
Anti-Sm (U/mL)	41	<7
Complement C3 (mg/dL)	44	80-120

**Figure 2:**
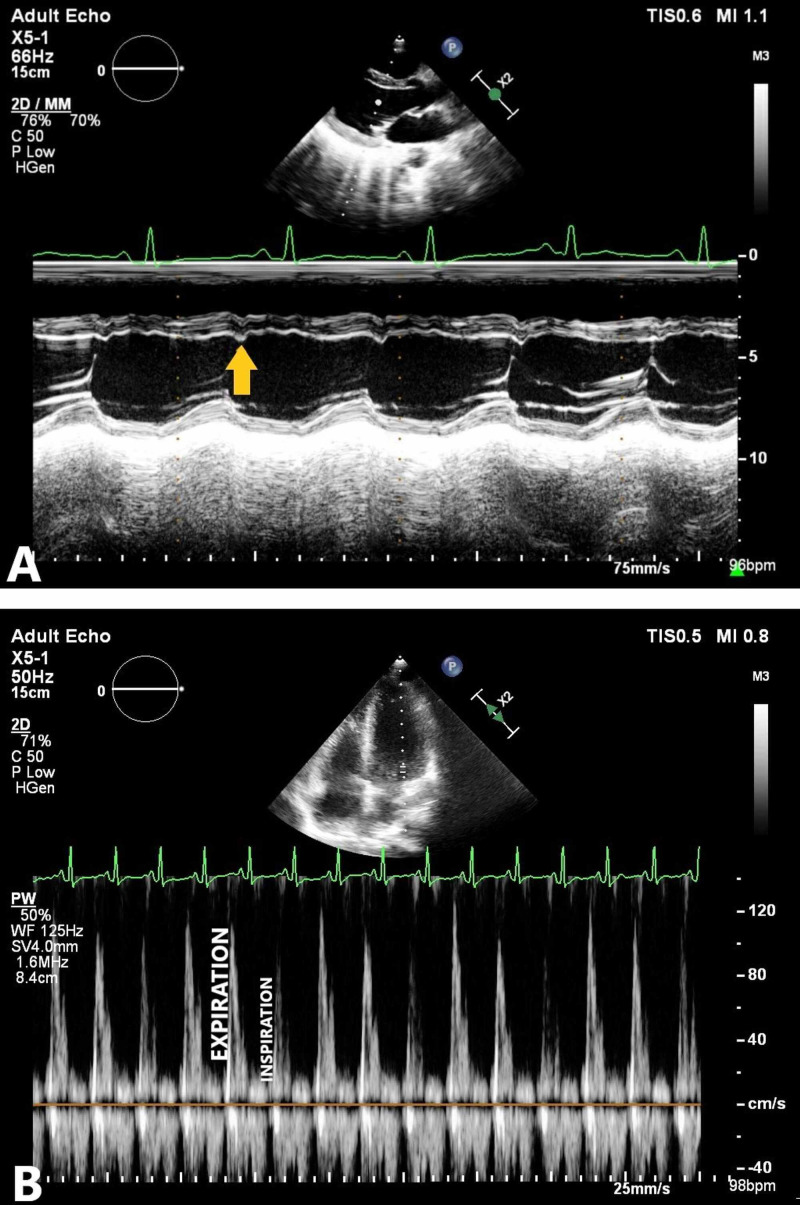
A) M-mode echocardiogram recording showing abnormal motion of the ventricular septum with notching (yellow arrow) in the early diastole, representing the diastolic dip. B) Pulsed-wave doppler spectrum of mitral inflow velocities demonstrating marked respiratory variation (more than 25%) of peak E-wave velocity.

**Figure 3:**
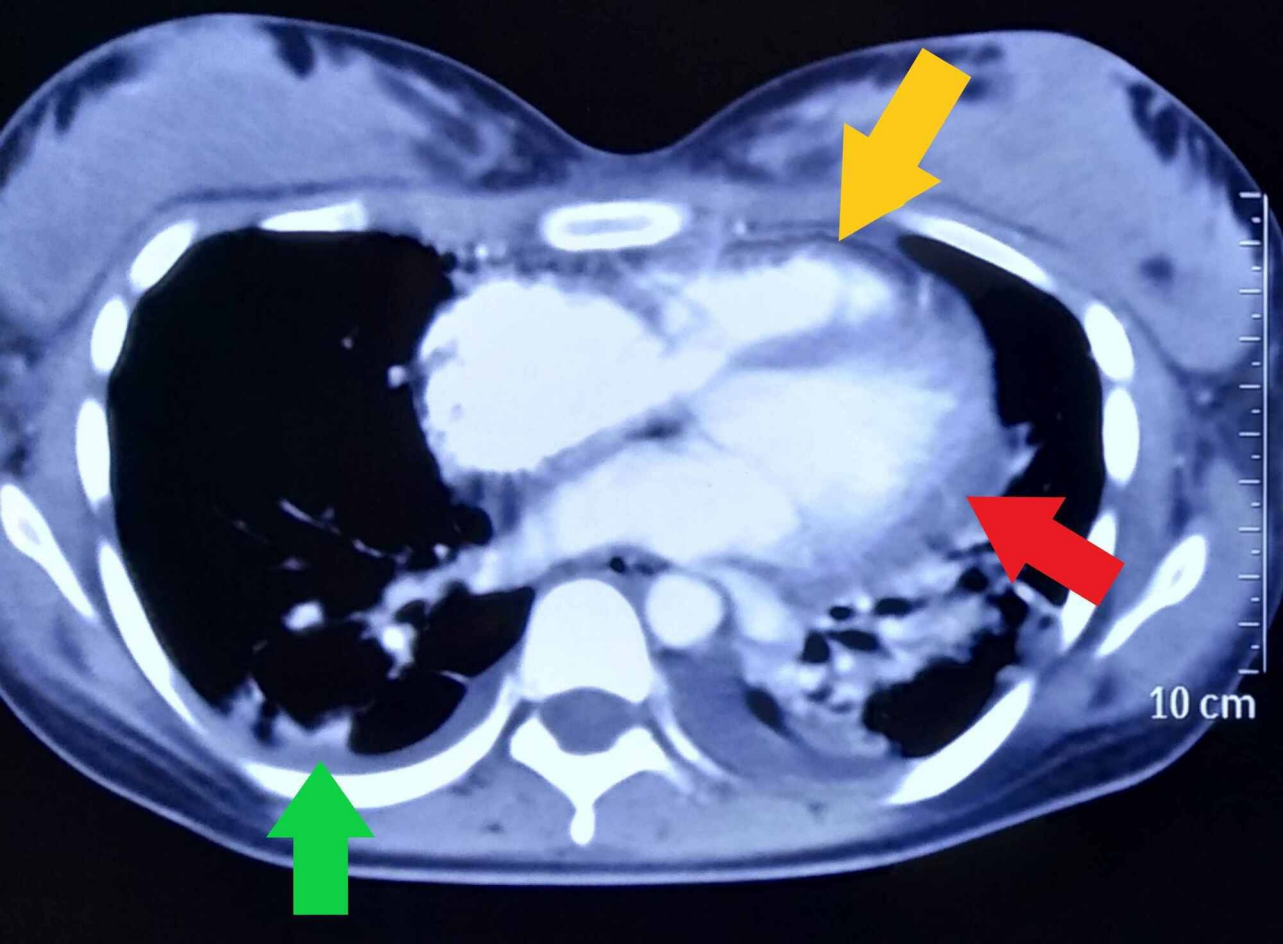
Computed tomographic scan of chest showing thickened pericardium (yellow arrow), pericardial effusion (red arrow) and pleural effusion (green arrow).

**Figure 4:**
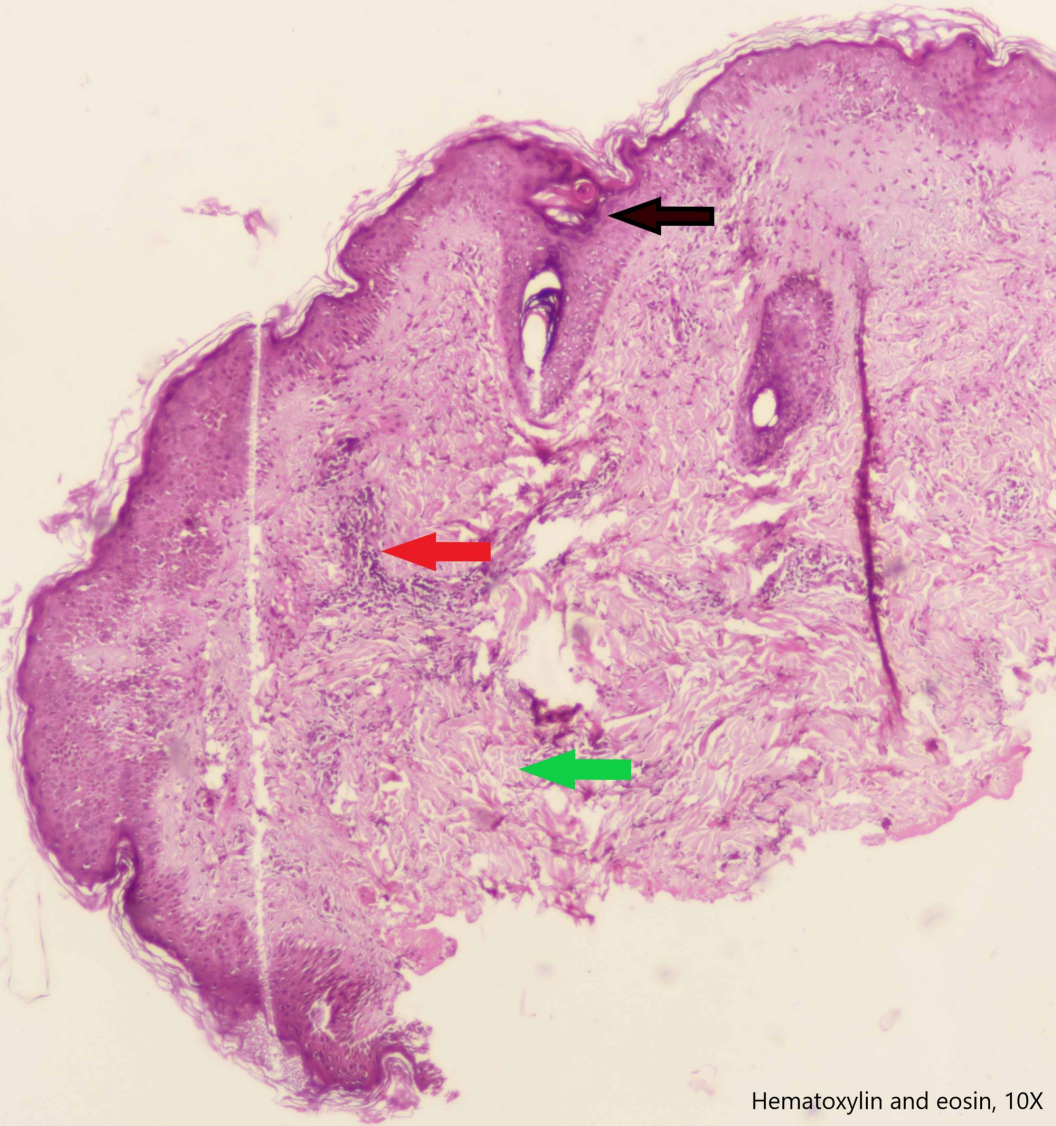
Skin biopsy showing focal follicular plug (black arrow), lymphomononuclear infiltrate (red arrow) and homogenization of collagen (green arrow).

Echocardiography showed a thickened pericardium with effusion, and motion abnormalities consistent with CP. On M-mode echocardiogram, there was a slight lag between the onset of electrical depolarization and the motion of interventricular septum and the posterior left ventricular wall. The peak downward motion of the interventricular septum slightly preceded the peak upward motion of the posterior wall. In early diastole, there was a small downward movement of the septum (diastolic dip) as a result of early right ventricular filling that preceded the filing of the left ventricle. This is illustrated in figure 2. There were no valvular lesions, and the systolic function was normal with a left ventricular ejection fraction of 60%. Computed tomographic scan (Figure 3) of the chest revealed pericardial effusion, pericardial thickening and normal lung parenchyma with mild bilateral pleural effusion. Analysis of the pleural effusion confirmed its sterile and transudative nature. Tuberculosis was ruled out by polymerase chain reaction, acid-fast staining and culture. Skin biopsy (figure 4), taken from her back, revealed changes consistent with SLE. These included plugging of the hair follicles with keratin, homogenization of collagen and moderate perivascular and peri-appendiceal lymphomononuclear infiltrate in the dermis.

The patient was administered 750 mg of intravenous methylprednisolone for three days, followed by prednisolone 40mg once a day. She was also given hydroxychloroquine, proton pump inhibitor, calcium and vitamin D supplements. Improvement was noticed during the course of admission in terms of dyspnea, rash and general well-being. A repeat echocardiogram after three weeks demonstrated normalized motion of the ventricular septum and reduced respiratory variation in E-wave velocity. Prednisolone was gradually tapered and stopped entirely after four months without any relapse of the disease.

## Discussion

A gamut of cardiac diseases can occur in connective tissue disorders. These include pericarditis, myocarditis, cardiomyopathy, arrhythmias, valvular dysfunction, coronary artery disease, thromboembolism and aneurysm formation [3]. Although all the three layers of the heart (endocardium, myocardium and pericardium) can be diseased in SLE, the most commonly reported cardiac manifestation is pericarditis [4]. In a study conducted on SLE patients without any clinically apparent heart disease, a multitude of echocardiographic abnormalities were found [5]. These included valvular lesions (47.5%), pericardial effusion (13.6%), pulmonary artery hypertension (8.5%), pericardial thickening (6.8%), impaired systolic function (3.4%) and left ventricular hypokinesia (1.7%). No abnormality was seen in 44.1% of the patients. A form of non-bacterial endocarditis, called Libman-Sacks endocarditis, can be seen in SLE. It is characterized by the formation of sterile verrucous vegetations on the valves that seldom embolize or produce valvular dysfunction [4].

A quarter of all patients with SLE develop clinical pericarditis during a lifetime, and the incidence of the asymptomatic disease is as high as 50% [4]. Cardiac tamponade and CP are very rare. The inflammatory process in pericardium is arbitrated by immune complexes and complement system activation, which leads to pericarditis [6]. Antibodies (IgG, IgA and IgM) and complement C3 have been detected in the pericardium [7]. Acute pericardial involvement is characterized by effusive or fibrinous changes and the insult, if prolonged and recurrent, progresses to scarring with development of CP. CP due to drug-induced lupus has also been reported secondary to procainamide and hydralazine [7,8]. CP as an initial manifestation of SLE is exceedingly rare. Lustig et al. reported it in a 30-year-old woman who presented with right-sided heart failure and chest pain [9]. She eventually required pericardiectomy. McMechan et al. have reported mixed effusive-constrictive pericarditis as an initial presentation of SLE in a 62-year-old woman who had developed cardiac tamponade and required surgical drainage [10].

CP in SLE is an indication for systemic corticosteroid therapy, and it should be instituted quickly after exclusion of infections [3,4]. Pericardial fluid, if present, may need to be aspirated and analyzed in cases with diagnostic uncertainty. Tamponade is an emergency and drainage should be done to avoid cardiovascular collapse. Acute pericarditis should be treated promptly with hydroxychloroquine, steroids and non-steroidal anti-inflammatory drugs to reduce the formation of pericardial adhesions and progression to CP. Recurrent pericarditis requires the addition of steroid-sparing agents, such as azathioprine and mycophenolate mofetil [11]. In advanced disease, diuretics are needed to optimize fluid status, and pericardiectomy is the definite treatment.

## Conclusions

CP is rare in SLE, and it generally results from repeated episodes of acute pericarditis. This novel report describes CP as an initial manifestation of SLE. The patient had presented with constitutional symptoms, the typical rash and dyspnea. Investigations revealed a lupus flare with pleural, pericardial and dermatological involvement. There was no evidence of any infection or an overlap syndrome. Treatment with corticosteroids was successful, and remission of the disease was achieved. Consideration should be given to SLE when the cause of CP is obscure as early diagnosis and treatment with immunosuppression can lead to a good outcome.
